# Short-Term Temperature Response of Leaf Respiration in Different Subtropical Urban Tree Species

**DOI:** 10.3389/fpls.2020.628995

**Published:** 2021-01-14

**Authors:** Man Xu, Lìyǐn L. Liáng, Miko U. F. Kirschbaum, Shuyi Fang, Yina Yu

**Affiliations:** ^1^College of Forestry and Landscape Architecture, South China Agricultural University, Guangzhou, China; ^2^Manaaki Whenua – Landcare Research, Palmerston North, New Zealand

**Keywords:** Leaf respiration, temperature response, temperature sensitivity, macromolecular rate theory, enthalpy, entropy, Gibbs free energy of activation

## Abstract

Plant leaf respiration is one of the critical components of the carbon cycle in terrestrial ecosystems. To predict changes of carbon emissions from leaves to the atmosphere under a warming climate, it is, therefore, important to understand the thermodynamics of the temperature response of leaf respiration. In this study, we measured the short-term temperature response of leaf respiration from five different urban tree species in a subtropical region of southern China. We applied two models, including an empirical model (the Kavanau model) and a mechanistic model (Macromolecular Rate Theory, MMRT), to investigate the thermodynamic properties in different plant species. Both models are equivalent in fitting measurements of the temperature response of leaf respiration with no significant difference (*p* = 0.67) in model efficiency, while MMRT provides an easy way to determine the thermodynamic properties, i.e., enthalpy, entropy, and Gibbs free energy of activation, for plant respiration. We found a conserved temperature response in the five studied plant species, showing no difference in thermodynamic properties and the relative temperature sensitivity for different species at low temperatures (<42°C). However, divergent temperature response among species happened at high temperatures over 42°C, showing more than two-fold differences in relative respiration rate compared to that below 42°C, although the causes of the divergent temperature response remain unclear. Notably, the convergent temperature response at low temperatures could provide useful information for land surface models to improve predictions of climate change effects on plant respiration.

## Introduction

Plants contribute a substantial amount of carbon to the atmosphere annually through respiration, about 60 Gt C per year (60 billion tons) with half of that coming from leaf respiration (Atkin et al., 2007). Understanding the temperature response of leaf respiration is, therefore, critical for predicting the carbon release from plants to the atmosphere under a warming climate. Application of models to explore the thermodynamic properties that regulate the temperature response of leaf respiration can provide useful information for understanding the dynamics of carbon release from leaf respiration.

The Arrhenius function is widely used to describe the temperature dependence of leaf respiration (Kruse et al., 2011; O’Sullivan et al., 2013), projecting an exponential increase in respiration rate with increasing temperature. However, the nonlinearity observed in Arrhenius plot (Kavanau, 1950; Lloyd and Taylor, 1994) could constrain the application of the Arrhenius function to accurately capture the temperature response of biological processes, including enzyme-catalyzed reactions (Hobbs et al., 2013; Arcus et al., 2016; Prentice et al., 2020), organism growth (Corkrey et al., 2014; Prentice et al., 2020) and ecophysiological processes like leaf respiration (O’Sullivan et al., 2013; Heskel et al., 2016; Liang et al., 2018) and soil respiration (Lloyd and Taylor, 1994; Schipper et al., 2014).

In an early work on analyzing the temperature response of biological processes that are regulated by enzymes, Kavanau (1950) proposed an empirical function to describe the curvature of the Arrhenius plots, and provided a way to calculate thermodynamic properties, including enthalpy, entropy, heat capacity, and free energy of activation. The curvature of the Arrhenius plots can also be captured by using a 2nd-order polynomial or a modified Arrhenius function (Kruse et al., 2011; Heskel et al., 2016), although the derived parameters have limited biological or thermodynamic meanings. Recent theoretical development on the temperature dependence of enzyme catalyzed reactions proposed a relatively simple model, i.e., macromolecular rate theory (MMRT) (Hobbs et al., 2013; Arcus et al., 2016), to capture the curvature and enable determination of thermodynamic properties. MMRT ascribed the observed curvature in the Arrhenius plots to the reduction of heat capacity between the ground state and transition state of the enzyme-substrate complex. Thermodynamic properties, like entropy, heat capacity and Gibbs free energy of activation, play important roles in controlling the temperature response (Karplus, 2000; Zhao, 2012). It is, therefore, important to apply appropriate models to explore thermodynamic properties in ecophysiological processes like leaf respiration for accurately predicting the impacts of the warming climate on leaf respiration.

Among those models, MMRT is equivalent to the 2nd-order polynomial in fitting the temperature response of leaf respiration (Liang et al., 2018) and presumably is equivalent to the Kavanau (1950) model. In this paper, we measured the short-term temperature response of leaf respiration of five different urban tree species and implemented two models, i.e., the Kavanau empirical model and MMRT, to investigate the thermodynamic properties of leaf respiration, including enthalpy, entropy, heat capacity and free energy of activation. We compared the performance of two models in describing the temperature response of leaf respiration. We further investigated the response of leaf respiration to temperature for different plant species. Results from this study could improve understanding of the thermodynamic properties that control leaf respiration and the climate warming effects on leaf respiration.

## Materials and Methods

### Plant Material and Gas Exchange Measurements

For this experiment, we used five urban tree species, i.e., *Ficus virens* Ait., *Ficus altissima* BL., *Elaeocarpus apiculatus* Masters in Hook., *Michelia × alba* DC., and *Cinnamomum burmannii* (C. G. et Th. Nees) Bl. The selection of species was based on a preliminary experiment where we sampled a wider range of species to find species with different specific leaf areas (SLAs). For the gas exchange measurements, we used one-year-old seedlings raised from seeds in a plant nursery. We conducted the gas exchange measurement experiments in the laboratory of the College of Forestry and Landscape Architecture at the South China Agricultural University, Guangzhou in the winter of 2019. Before taking measurements of leaf respiration, each plant was put in darkness for about 30-min to allow adaption. We then selected mature, healthy, and fully expanded leaves to take gas exchange measurements.

Dark respiration over a range of temperatures was determined using a gas exchange measuring system (GFS3000, Walz, Effeltrich, Germany). The sampled leaf was put into a 3 cm^2^ cuvette which was flushed with air flowing at a rate of 400 μmol m^–2^ s^–1^ and 380 ppm CO_2_ under a relative humidity of ∼60% at around 20°C. The chamber temperature was controlled by the system with a preset program, that could change the temperature up to 50°C. The chamber was heated at a rate of 1°C min^–1^, and respiration rate was recorded every 30 s. After gas exchange measurements, we excised the sampled leaves and measured their leaf area using a handheld laser leaf area meter (CI-203, CID Bio-Science Inc.). Subsequently, leaves were put in a drying cabinet at 65°C for about 72 h before being weighed to determine their dry mass and to calculate specific leaf area (m^2^ kg^–1^). The gas exchange and SLA measurements included four to five replicates from different plants for each species, with 23 samples in total.

### Descriptions of the Kavanau Model and MMRT

**FIGURE 1:**
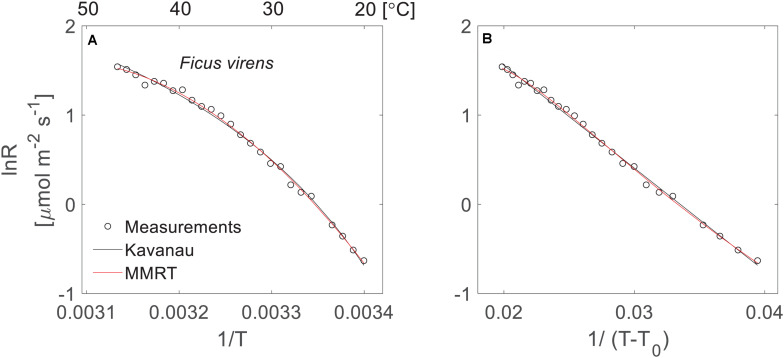
Temperature response of leaf respiration under different scales of *x*-axis. **(A)** The non-linearity of the Arrhenius plot using the logarithm of leaf respiration rate (*lnR*) of *F. virens* (black circles) plotted against *1/T* with temperature expressed in Kelvins. The black and red lines show model fits to the measurements using the Kavanau model and MMRT, respectively. **(B)** The linearisation of the temperature response curve using the same data from panel **(A)**, but the *x*-axis was changed from *1/T* to *1/(T-T_0_)*, where *T*_0_ = 268.8 K.

Kavanau (1950) proposed an empirical model to describe the curvature of the temperature response curves in Arrhenius plots as:

(1) $$k=de^{-\frac{E_{0}}{T-T_{0}}}$$

where *k* is the rate, *d* and *E*_0_ are empirical fitted parameters. *T*_0_ is the temperature (Kelvin) at which the rate is zero and *T* is the absolute temperature (Kelvin). In this equation, the parameter *E*_0_ has no biological meaning and is completely different from the activation energy term of the Arrhenius equation. This empirical model aims to linearise temperature response curves. When *lnk* empirically are plotted against *1/T* (in conformity with the Arrhenius relationship), it results in non-linear relationships for biological processes (Kavanau, 1950). Plotting *lnk* against *1/(T-T_0_)* can turn it into a more linear relationship by changing the scale in *x*-axis (Whittemore, 1923). See an example in Figures 1A,B. Based on further analysis of kinetic theory, Kavanau (1950) pointed out that this empirical model is an approximation of the exact form, which is described by an incomplete gamma function of 3/2 and *E_0_/(T–T_0_)*. However, it is unclear whether *E_0_/(T–T_0_)* will follow gamma distribution as *E_*a*_/RT* in the Arrhenius equation. In the present study, we used Eq. 1 as an empirical model and derived the corresponding thermodynamic properties, i.e., enthalpy, entropy and heat capacity, following Kavanau (1950) by using the Eyring equation.

The Eyring equation describes the temperature response of a reaction as

(2) $$k=\frac{{\text{k}}_{B}T}{h}e^{-\frac{\Delta G^{‡}}{RT}}=\frac{{\text{k}}_{B}T}{h}e^{-\frac{\Delta H^{‡}-T\Delta S^{‡}}{RT}}$$

where the *k*_*B*_, *h*, and *R* are Boltzmann, Planck and ideal gas constants, respectively. Δ*H*^‡^ and Δ*S*^‡^ are the change in enthalpy and entropy between the ground state and the transition state of a reaction, respectively. Δ*H*^‡^ and Δ*S*^‡^ further determine Δ*G*^‡^, the Gibbs free energy at a reference temperature *T* as Δ*G*^‡^ = Δ*H*^‡^ – *T*Δ*S*^‡^.

By comparing the slopes of the *lnk ∼1/T* plots from Eqs. 1 and 2 (see details in Supplementary Materials), we can get

(3) $$\Delta H^{‡}=\frac{E_{0}RT^{2}}{{\left(T-T_{0}\right)}^{2}}-RT$$

The heat capacity is then determined by the derivative of Δ*H*^‡^ based on Eq. 3 as

(4) $$\Delta C_{p}^{‡}=\frac{d\Delta H^{‡}}{dT}=\frac{2E_{0}RT}{{\left(T-T_{0}\right)}^{2}}-\frac{2E_{0}RT^{2}}{{\left(T-T_{0}\right)}^{3}}-R$$

The change in entropy, Δ*S*^‡^, can then be calculated from Eq. 2 using the predicted rate in Eq. 1. It should be noted that the heat capacity increase with increasing temperature in Eq. 4. Within the biological temperature range, the temperature dependence of heat capacity could be small, assuming no change in Δ*C*_*p*_^‡^. Therefore, Eq. 2 can be re-arranged to form MMRT by introducing a reference temperature (*T*_*ref*_) to determine the reference enthalpy $\left(\Delta H_{T_{ref}}^{‡}\right)$ and entropy $\left(\Delta S_{T_{ref}}^{‡}\right)$. MMRT has the form of

(5) $$\begin{array}{l} k=\frac{{\text{k}}_{B}T}{h}e^{-\frac{\Delta H^{‡}-T\Delta S^{‡}}{RT}}\\ =\frac{{\text{k}}_{B}T}{h}e^{-\frac{\Delta H_{T_{ref}}^{‡}+\Delta C_{P}^{‡}\left(T-T_{ref}\right)-T\left(\Delta S_{T_{ref}}^{‡}+\Delta C_{P}^{‡}\left(\text{ln}T-\text{ln}T_{ref}\right)\right)}{RT}} \end{array}$$

In MMRT, the way to calculate Δ*H*^‡^ and Δ*S*^‡^ is more straightforward compared to the empirical Kavanau model, as$\Delta H^{‡}=\Delta H_{T_{ref}}^{‡}+\Delta C_{P}^{‡}\left(T-T_{ref}\right)$ and $\Delta S^{‡}=\Delta S_{T_{ref}}^{‡}+\Delta C_{P}^{‡}\left(\text{ln}T-\text{ln}T_{ref}\right)$.

Except for the thermodynamic properties, MMRT also provides a way to calculate the temperature optimum (*T*_*opt*_) at which the rate is the highest and the inflection point (*T*_*inf*_) at which the rate is most sensitive to changes in temperature. Both *T*_*opt*_ and *T*_*inf*_ can be derived from Eq. 5 as

(6) $$T_{\text{opt}}=\frac{\Delta H_{T_{0}}^{‡}-\Delta C_{p}^{‡}T_{0}}{-\Delta C_{p}^{‡}-R}\mathrm{and}T_{\text{inf}}=\frac{\Delta H_{T_{0}}^{‡}-\Delta C_{p}^{‡}T_{0}}{-\Delta C_{p}^{‡}\pm \sqrt{-\Delta C_{p}^{‡}R}}$$

Similarly, we can derive the *T*_*inf*_ from the Kavanau model based on Eq. 1 as (see the derivation in Supplementary Material):

(7) $$T_{\text{inf}}=T_{0}+0.5E_{0}$$

but Eq. 1 has no *T*_*opt*_.

### Curve Fitting and Statistical Analysis

We fitted the measured temperature response curves (23 curves in total) to the Kavanau model and MMRT, respectively, and further calculated the thermodynamic properties based on the parameter estimates from MMRT. The respiration data (R) were log-transformed before fitting. We used the *nlinfit* function of MATLAB and Statistics Toolbox Release (2018a) to fit *lnR∼1/T* curve (*lnR* is the logarithm of leaf respiration rate, equivalent to the logarithm of *k* in Eq. 1 as *lnk*. We used *lnR* to denote the respiration rate hereafter). For MMRT, the reference temperature (*T*_*ref*_) was set to 298.15 K. For comparison of model performance, we calculated the Nash and Sutcliffe model efficiency (NSE) (Nash and Sutcliffe, 1970) for each *lnR∼1/T* curve when we fitted MMRT and the Kavanau model to the measurements. A *t*-test was used to check the difference in NSE between MMRT and the Kavanau model. We applied one-way ANOVA to test the differences in thermodynamic properties over different species and SLAs. Statistical analyses, model fittings and data processing were performed using MATLAB R2018a (The MathWorks Inc., Natick, MA, United States).

## Results and Discussion

### Model Performances

**FIGURE 2:**
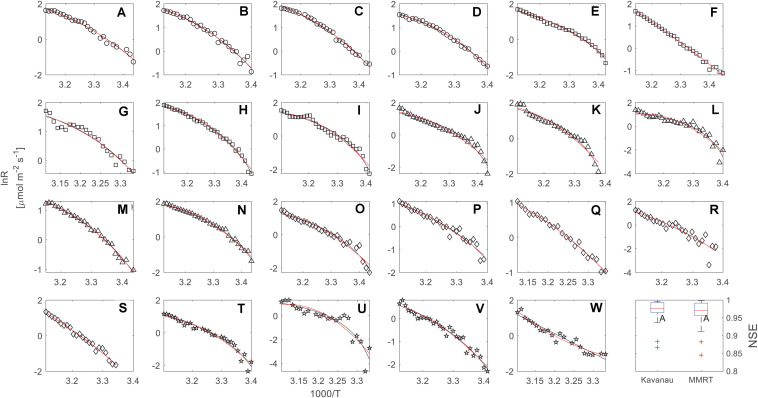
Temperature response measurements (black circles) of leaf respiration over five different urban tree species, including *F. virens* (circles, **A–D**), *F. altissima* (squares, **E–I**), *M. alba* (triangles, **J–N**), *E. apiculatus* (diamonds, **O–S**), and *C. burmannii* (pentagrams, **T–W**). Each curve (23 curves in total) was fitted by the Kavanau model (black line) and MMRT (red line). The last panel gives a comparison of Nash and Sutcliffe model efficiency (NSE) between the Kavanau model and MMRT for 23 the individual temperature response curves.

In our dataset, both the Kavanau model and MMRT could be applied successfully to describe the temperature response of leaf respiration, and captured the curvature of the temperature response curves (Figure 2). The Kavanau model empirically reduces the curvature in the *lnR∼1/T* plot by scaling the *x*-axis from *1/T* to *1/(T–T_0_)*. Taking the temperature response curve of *F. virens* as an example, the curvature is apparent when *lnR is* plotted against *1/T* (Figure 1A) but it can be reduced when *lnR* is plotted against *1/T–T_0_* (Figure 1B). Mathematical manipulations that result in linearisation can be useful for curve fitting, but biological information can be lost through this manipulation. In contrast, in MMRT, the curvature is due to a reduction in heat capacity of the enzyme-substrate complex from the ground state to the transition state (Hobbs et al., 2013; Arcus et al., 2016), providing insightful information for the understanding of enzyme-catalyst reactions for leaf respiration. Nevertheless, both models provide similarly good agreement with observed leaf respiration across the range of measurement temperatures and different species (Figure 2), with no difference in NSE (*p* = 0.67).

### Thermodynamic Properties

When *lnR* was plotted against *1/T* in our dataset of 23 individual temperature response curves, we commonly observed non-linear relationships (Figure 2). This curvature seems to be a generic feature of the temperature response curve observed in many biological processes across different scales, including enzyme-catalyst reactions (Hobbs et al., 2013; Arcus et al., 2016), organism growth (Corkrey et al., 2012; Prentice et al., 2020), leaf respiration (Kruse and Adams, 2008; O’Sullivan et al., 2013; Heskel et al., 2016; Liang et al., 2018; Inoue and Noguchi, 2020) and soil respiration (Schipper et al., 2014, 2019; Robinson et al., 2017).

All these biological processes are related to enzymatic reactions. At the enzyme scale, the decrease of heat capacity of the enzyme-substrate complex from the ground state to the active state is believed to be the cause for the curvature in the temperature response measurements for enzyme-catalyzed reactions (Arcus et al., 2016). The change in heat capacity is the most distinguished character of MMRT compared to the Arrhenius function or Eyring equation that assumes no change in heat capacity i.e., Δ*C*_*p*_^‡^ = 0, thus temperature independence of Δ*H*^‡^ and Δ*S*^‡^. However, evidence from experimental works and protein simulations [see details on a recent review by Arcus and Mulholland (2020)] suggests the temperature dependence of Δ*H*^‡^ and Δ*S*^‡^, therefore further infers a change in Δ*C*_*p*_^‡^ for enzyme-catalyzed reactions. One of the consequences of the temperature dependence of Δ*H*^‡^ and Δ*S*^‡^ is the nonlinear change of Δ*G*^‡^ with temperature, departing from the linear relationship, i.e Δ*G*^‡^ = Δ*H*^‡^ – TΔ*S*^‡^ proposed in the Eyring equation or a constant activation energy with temperature in the Arrhenius function.

**FIGURE 3:**
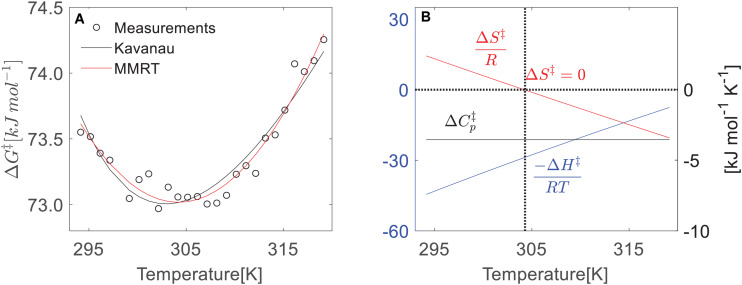
Temperature dependence of thermodynamic properties (Δ*H*^‡^, Δ*S*^‡^, and Δ*G*^‡^) of leaf respiration from *F. virens*. **(A)** The temperature dependences of Δ*G*^‡^ that is determined by the absolute rate function Eq. 2 based on the measurements. Both the Kavanau (black line) and MMRT (red lin e) can predict the change of Δ*G*^‡^. **(B)** Temperature dependence of Δ*H*^‡^ and Δ*S*^‡^ from MMRT, assuming the temperature independence of Δ*Cp*^‡^ as a constant.

Indeed, we did find the nonlinear change of Δ*G*^‡^ from our leaf respiration measurements. Taking the data from *F. virens* shown in Figure 1 as an example, we showed a strong nonlinearity of Δ*G*^‡^ across the measured temperatures (Figure 3A). In the Arrhenius function, the activation energy is assumed to be temperature independent. However, this assumption could be violated for describing the temperature response of leaf respiration because of the strong temperature dependence of Δ*G*^‡^ (Figure 3A), which is well described by both the Kavanau model and MMRT. Compared with the Eyring equation or the Arrhenius function, the Kavanau model or MMRT provide a way to describe the curvature observed in the temperature dependence of leaf respiration. Therefore, application of the Kavanau model or MMRT in land surface models could enable more precise prediction of the carbon release from leaf respiration in a warming climate.

The changes in enthalpy (Δ*H*^‡^) and entropy (Δ*S*^‡^) with temperature in the temperature response of leaf respiration is similar to that in enzyme-catalyst reactions (Arcus et al., 2016), showing decreasing Δ*H*^‡^ and Δ*S*^‡^ with increasing temperature (Figure 3B) using MMRT. As we have pointed out in Method section, the Kavanau model and MMRT describe the temperature dependence of Δ*H*^‡^, Δ*S*^‡^, and Δ*C*_*p*_^‡^ in different ways. In the Kavanau model, Δ*H*^‡^ and Δ*S*^‡^ decrease non-linearly with increasing temperature while Δ*C*_*p*_^‡^ increases. In MMRT, the temperature dependence of Δ*H*^‡^ and Δ*S*^‡^ is almost linear while Δ*C*_*p*_^‡^ is temperature independent. Answering the question of whether Δ*C*_*p*_^‡^ is temperature independent, requires further investigation from biochemistry (Arcus and Mulholland, 2020) and it is beyond the scope of this study. Despite this difference, both models describe the temperature dependence of Δ*G*^‡^ similarly (Figure 2A), thus provide equivalent estimates on the short-term temperature response of leaf respiration.

Besides, a discrepancy is observed between the Kavanau model and MMRT in estimating the *T*_*inf*_, a meaningful physiological parameter of leaf respiration. We find the *T*_*inf*_ estimated by the Kavanau model is often higher than that of from MMRT (Supplementary Figure 1). The Kavanau model seems to provide unrealistic estimates on *T*_*inf*_ for some cases (Supplementary Figure 1), such as over 100°C that is far beyond the biological relevant temperature. The insufficiency of the Kavanau model in determining *T*_*inf*_ may lie in the incomplete linearisation of the curvature by scaling the *x-*axis from *1/T* to *1/T–T_0_*, resulting in inaccurate estimates in both E_0_ and T_0_. Therefore, we use the derived thermodynamic properties and *T*_*inf*_ from MMRT in the following analysis.

### Temperature Response and Thermodynamic Properties for Different Species

**FIGURE 4:**
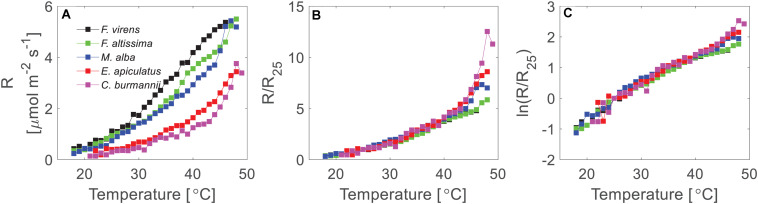
Temperature response of leaf respiration of five urban tree species. **(A)** Demonstrated the mean short-term temperature response measurements on a linear scale. **(B)** Demonstrated the normalized temperature response curves for five different species. The rates were normalized to the rates at 25°C of each species. **(C)** Showed the normalized rates at a log-scale. The mean respiration rates were calculated where at least two measurements (two replicates) were available at each temperature for each species.

Leaf respiration varied considerably between the five different studied species, with the highest respiration rates in *F. virens* and the lowest in *C. burmannii* (Figure 4A). For example, at 25°C, the mean respiration rate of *F. virens* was 1.01 μmol m^–2^ s^–1^ (Table 1), which was about five times as high as the rate of *C. burmannii* (0.19 μmol m^–2^ s^–1^). Interestingly, the relative temperature sensitivity, e.g., Q_10_ at 25°C, was remarkably conserved, with no differences between species (Table 1), showing a similar shape of the response curves for five different species (Figures 4B,C). A clearer picture of this conserved response pattern can be seen by normalizing the mean respiration rate to the reference temperature at 25°C of each species (Figures 4B,C), showing nearly overlapped temperature response curves below 42°C for all species. Above 42°C measured rates diverged strongly, with relative rates at 48°C ranging more than two-fold while there were no differences in relative rates below 42°C.

**TABLE 1:** Means and standard errors of specific leaf area (SLA), the changes in enthalpy $\left(\Delta H_{T_{ref}}^{{}^{‡}}\right)$, entropy $\left(\Delta S_{T_{ref}}^{‡}\right)$, heat capacity $\left(\Delta C_{p}^{{}^{‡}}\right)$, the inflection temperature (*T*_*inf*_), respiration rate (R) and the relative temperature sensitivity (Q_10_) at *T*_*ref*_ (*T*_*ref*_ = 298.15 K or 25°C) from MMRT of five species.

Tree species	SLA	$\Delta H_{T_{ref}}^{‡}$	$\Delta S_{T_{ref}}^{‡}$	$\Delta C_{P}^{‡}$	*T*_*inf*_	*R* at 25°C	Q_10_ at 25°C
	[m^2^ kg^–1^]	[kJ mol^–1^]	[kJ mol^–1^K^–1^]	[kJ mol^–1^ K^–1^]	[°C]	[μ mol m^–2^ s^–1^]	
*F. virens*	12.7 ± 0.5^a^	93.4 ± 3.3^a^	0.07 ± 0.01^a^	−3.1 ± 0.2^a^	39.5 ± 1.4^a,b^	1.01 ± 0.11^a^	2.9 ± 0.1^a^
*F. altissima*.	10.3 ± 0.6^a*^	110.8 ± 10.4^a^	0.12 ± 0.03^a^	−4.1 ± 0.5^a^	38.1 ± 0.9^a,b^	0.75 ± 0.18^a,b^	3.4 ± 0.4^a^
*M. alba*	11.7 ± 0.5^a^	117.7 ± 13.6^a^	0.15 ± 0.04^a^	−4.6 ± 0.6^a^	37.7 ± 1.1^a^	0.66 ± 0.14^a,b^	3.7 ± 0.5^a^
*E. altissima*	11.5 ± 0.3^a^	96.1 ± 5.6^a^	0.07 ± 0.02^a^	−2.7 ± 0.5^a^	43.9 ± 2.6^*b*^	0.43 ± 0.06^a,b^	3.1 ± 0.1^a^
*C. burmannii*	12.9 ± 1.1^a^	190.6 ± 80.7^a^	0.38 ± 0.26^a^	−3.6 ± 0.6^a^	40.3 ± 0.7^a,b^	0.19 ± 0.09^*b*^	3.5 ± 0.3^a^
*p*-value	0.055	0.23	0.25	0.26	0.06	0.013	0.52

Values were not significantly different (significance level of α = 0.05) across species with the same letters within columns.

*marginally significant (0.05 ≤ *p* < 0.1).

*“a” or “b” are letters to denote the statistical difference.*

At low temperatures, the conserved temperature response suggested similar thermodynamic properties of respiratory enzymes for different plant species, since respiration rate at low temperatures is mainly regulated by the capacity of respiratory enzymes (Atkin et al., 2000, 2005; Covey-Crump et al., 2002; Atkin and Tjoelker, 2003). Further comparisons on the thermodynamic properties showed that the change in *$\Delta H_{T_{ref}}^{{}^{‡}}$*, *$\Delta S_{T_{ref}}^{‡}$*, and Δ*Cp*^‡^ were indeed conserved, with no significant difference for different species (Table 1). The conserved pattern of temperature response and thermodynamic properties in leaf respiration has also been reported from a dataset including species across different biomes and plant function types at the global scale (Heskel et al., 2016; Liang et al., 2018). Our results support the notion that different plant species use similar enzymes in the respiration pathways, resulting in conserved thermodynamic properties and temperature response patterns at low temperatures.

At higher temperatures, a “burst” of leaf respiration rate has been observed previously (O’Sullivan et al., 2013). The divergent temperature response of leaf respiration could be affected by many factors. For example, factors could be phloem loading to export previously fixed carbohydrates from photosynthesis. Phloem loading requires energy as ATP from respiration (Thornley and Cannell, 2000). In this case, the rates and duration of previous photosynthesis could be important before the dark adaption, as well as the duration of the temperature response measurement of respiration. In our experiment, the respiration rate is measured with 1 and 2 min at each temperature, suggesting that these rates have little time to reflect an indirect response to these conditions. The divergent temperature response could also be regulated by the physiological processes of leaf respiration itself, such as changes in cell membrane properties (Schrader et al., 2004; Zhu et al., 2018), uncoupled from mitochondrial electron transport (Hüve et al., 2011), or drought stress at high temperatures (Atkin and Macherel, 2009). At high temperatures, the respiratory enzymes could be still functioning since they probably have higher temperature optima (Liang et al., 2018), while the organelle in leaf as mitochondria could be less resistant to high temperatures since the membrane is mainly consisted of fatty acid, which will likely become unstable at high temperatures. Revealing the temperature tolerance of leaf respiration among different species is worth further investigating in the subtropical region.

While relative temperature responses and thermodynamic properties were conserved, absolute rates differed between plant species. For example, the difference in SLA between *F. virens* and *C. burmannii* was small, 12.7 versus 12.9 m^2^ kg^–1^ (Table 1), while the difference in their rates at 25°C was significant (Table 1). The difference in SLA between *F. virens* and *F. altissima* was considerable, 12.7 versus 10.3 m^2^ kg^–1^, while their rates at 25°C were similar (Table 1 and Figures 4A,B). Regulations of respiration at the leaf level are sophisticated, and many factors could contribute to the control of actual respiration rates. Previous studies that aimed to determine universal scaling relationships between respiration and leaf traits came to different conclusions (e.g., Reich et al., 1998; Atkin et al., 2015; Rowland et al., 2017). Leaf nitrogen concentration, specific leaf area and/or leaf life-span have been identified as powerful plant traits affecting the relationships between leaf respiration and plant traits across large biogeographical scales (Reich et al., 1996, 1997, 1998; Atkin et al., 2015; Rowland et al., 2017). However, such scaling relationship was not found in our study. The lack of detecting a correlation between respiration rate and SLA could be due to the narrow range of SLA of the five tree species in our study.

Lastly, the conserved patterns of the temperature response and thermodynamic properties of leaf respiration for the five different species in our study agree with the MMRT expectation that the thermal response of respiratory enzymes is conserved (Arcus et al., 2016; Liang et al., 2018), showing no difference in thermal properties, i.e., enthalpy $\left(\Delta H_{T_{r}ef}^{‡}\right)$, entropy $\left(\Delta S_{T_{ref}}^{‡}\right)$, heat capacity $\left(\Delta C_{p}^{‡}\right)$ etc., of the corresponding enzymes across species (Table 1). This further implies a conserved relative temperature sensitivity (Q_10_, Table 1) and relative temperature response of the enzymatic processes in leaf respiration across five plant species with increasing temperature (Figure 4). Incorporating knowledge from short-term temperature response of respiration with long-term temperature acclimation information into land surface models could be useful to improve prediction of climate change effects on plant respiration (Gifford, 2003; Huntingford et al., 2017).

## Conclusion

We measured the short-term temperature response of leaf respiration in five different urban tree species native to the subtropical region of southern China. All our measured leaves showed nonlinearity of the temperature response in leaf respiration in the Arrhenius plot (*lnR∼1/T* plot), and the curvature in the Arrhenius plot could be captured by both the Kavanau model and MMRT. MMRT provides a straightforward way to calculate the enthalpy, entropy and Gibbs free energy of activation for plant respiration. We found that there is no difference in the derived thermodynamic properties from MMRT of leaf respiration in the investigated plant species. At temperatures below about 42°C, the temperature response pattern of leaf respiration was very similar for different species, showing no difference in the relative temperature sensitivity. However, the absolute respiration rate and temperature sensitivity did differ for five plant species. At high temperatures (above 42°C), the temperature response of leaf respiration for different species started to depart from the conserved pattern that happens at low temperatures (below 42°C), with more than two-fold differences in relative respiration rate at the highest measured temperatures compared to that below 42°C. The convergent temperature response and the associated derived thermodynamic properties could provide useful information to predict the warming effects on plant respiration by incorporating the long-term temperature acclimation data into land surface models. It also worth further investigating the causes for the divergent temperature response of leaf respiration at high temperatures to understand the temperature tolerance of different species under extreme climate events as heat-wave attacks.

## Data Availability Statement

The raw data supporting the conclusions of this article will be made available by the authors, without undue reservation.

## Author Contributions

MX, LL, MK, and YY conceived this article. MX and SF collected the data. LL wrote the draft with contribution from MK and MX. LL and MX did the data analysis. LL conducted the theoretical analysis in thermodynamics. All authors revised the manuscript and approved for submission.

## Conflict of Interest

The authors declare that the research was conducted in the absence of any commercial or financial relationships that could be construed as a potential conflict of interest.

## Supplementary Material

Supplementary Figure 1 Comparison of estimated *T*_*inf*_ between the Kavanau model and MMRT. **(A)** showed all estimated *T*_*inf*_ from the Kavanau model and MMRT without constraints for the realistic temperature range in *T*_*inf*_. More than half of *T*_*inf*_ estimates from Kavanau model sits outside the realistic range while MMRT provides better estimates on *T*_*inf*_. **(B)** compared the *T*_*inf*_ estimates from the Kavanau model and MMRT within the realistic range (<50°C).

Supplementary Figure 2 The temperature dependences of Δ*G*^‡^ in leaf respiration across five urban species including *F. virens* (circles, **A–D**), *F. altissima* (squares, **E–I**), *M. alba* (triangles, **J–N**), *E. apiculatus* (diamonds, **O–S**), and *C. burmannii* (pentagrams, **T–W**). Δ*G*^‡^ is determined by the absolute rate function Eq. 2 based on the measurements for each temperature response curve (23 in total). Both the Kavanau (black line) and MMRT (red line) can predict the change of Δ*G*^‡^. MMRT only predict the Δ*G*^‡^ when Δ*C*_*p*_^‡^ < 0, where the last measurement (panel w) has Δ*C*_*p*_^‡^ > 0 thus no valid Δ*G*^‡^ prediction from MMRT.
