# Supplementary material for: Short-Term Temperature Response of Leaf Respiration in Different Subtropical Urban Tree Species
Source: Front Plant Sci. 2021 Jan 14;11:628995. doi: 10.3389/fpls.2020.628995 (PMC7841330; doi:10.3389/fpls.2020.628995)

**Supplementary materials**

The slope of Eq. 1 in the plot of *lnk~1/T* is given by

$$\frac{dlnk}{d(\frac{1}{T})}=-\frac{E_0T^2}{(T-T_0)^2} \qquad (S1)$$

For Eq. 2,

$$\frac{dlnk}{d(\frac{1}{T})}=-\left(T+\frac{\Delta H^{\ddagger}}{R}\right) \qquad (S2)$$

From Eq. S1 and Eq. S2, we can obtain

$$\frac{E_0T^2}{(T-T_0)^2}=T+\frac{\Delta H^{\ddagger}}{R} \qquad (S3)$$

Re-arranging Eq. S3 gives

$$\Delta H^{\ddagger}=\frac{E_0RT^2}{(T-T_0)^2}-RT \qquad (3)$$

Derivation of $T_{inf}$ from Kavanau model

$$k=de^{-\frac{E_0}{T-T_0}} \qquad (1)$$

$$k'=\frac{dk}{dT}=de^{-\frac{E_0}{T-T_0}}\frac{E_0}{(T-T_0)^2}$$

$$k''=\frac{d^2k}{dT^2}=de^{-\frac{E_0}{T-T_0}}\frac{-E_0(2T-2T_0-E_0)}{(T-T_0)^4}$$

$T_{inf}$ is the temperature when $k''=0$, thus

$$2T_{inf}-2T_0-E_0=0$$

$$T_{\mathrm{inf}}=T_0+0.5E_0 \qquad (7)$$

Figure S1 Comparison of estimated $T_{inf}$ between the Kavanau model and MMRT. (a) showed all estimated $T_{inf}$ from the Kavanau model and MMRT without constraints for the realistic temperature range in $T_{inf}$. More than half of $T_{inf}$ estimates from Kavanau model sits outside the realistic range while MMRT provides better estimates on $T_{inf}$. (b) compared the $T_{inf}$ estimates from the Kavanau model and MMRT within the realistic range (<50 °C).

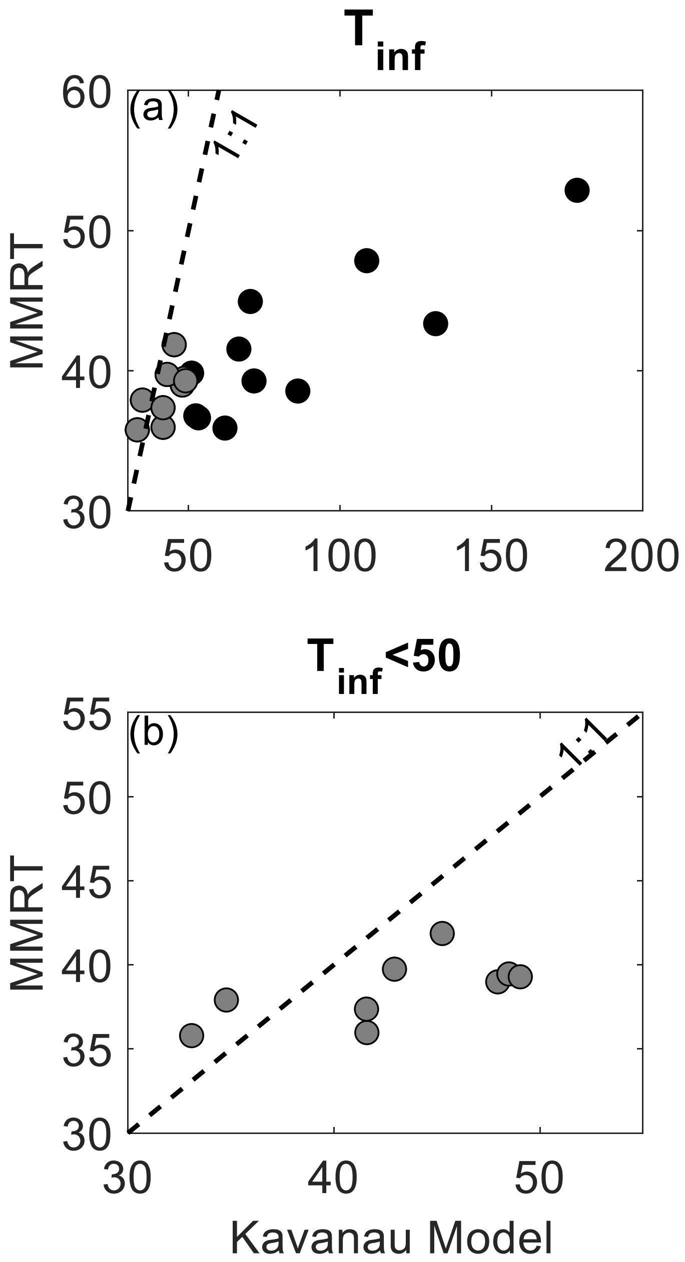

Figure S2 The temperature dependences of $\Delta G^{\ddagger}$ in leaf respiration across 5 urban species including *F. virens* (circles, A-D), *F. altissima* (squares, E-I), *M. alba* (triangles, J-N), *E. apiculatus* (diamonds, O-S), and *C. burmannii* (pentagrams, T-W). $\Delta G^{\ddagger}$ is determined by the absolute rate function Eq. 2 based on the measurements for each temperature response curve (23 in total). Both the Kavanau (black line) and MMRT (red line) can predict the change of $\Delta G^{\ddagger}$. MMRT only predict the $\Delta G^{\ddagger}$ when $\Delta C_p^{\ddagger}$<0, where the last measurement (panel w) has $\Delta C_p^{\ddagger}$>0 thus no valid $\Delta G^{\ddagger}$ prediction from MMRT.

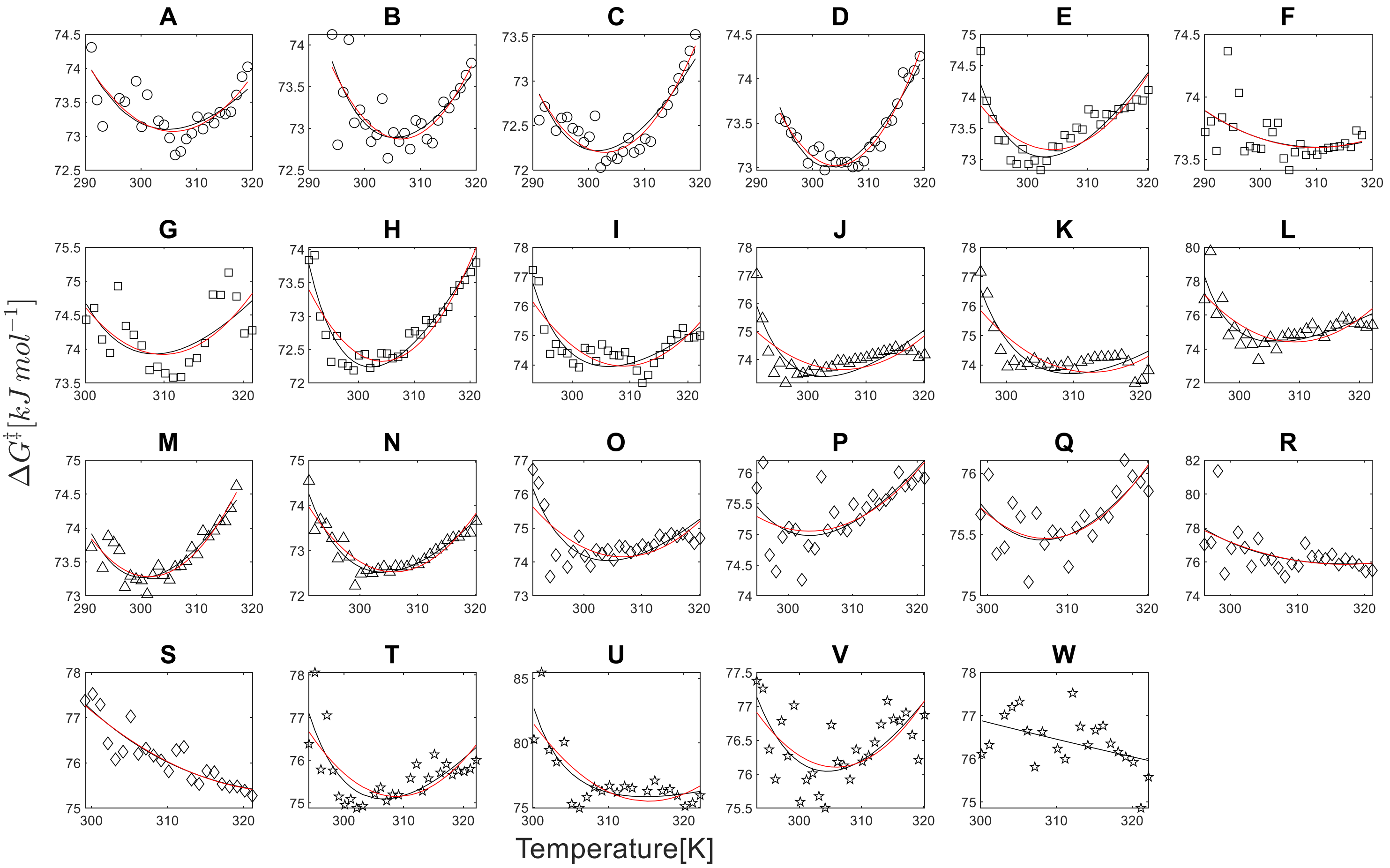

Supplement: Supplementary Figure 1 — Comparison of estimated Tinf between the Kavanau model and MMRT. (A) showed all estimated Tinf from the Kavanau model and MMRT without constraints for the realistic temperature range in Tinf. More than half of Tinf estimates from Kavanau model sits outside the realistic range while MMRT provides better estimates on Tinf. (B) compared the Tinf estimates from the Kavanau model and MMRT within the realistic range (<50°C). [file Presentation_1.pdf]
